# Case Report: 3D printing of customized combined guides assisting total hip arthroplasty for treatment of fused hip: a case report

**DOI:** 10.3389/fbioe.2025.1576305

**Published:** 2025-04-17

**Authors:** Xingyu Wang, Yifeng Jiang, Zhe Wang, Yu Zeng, Jiaxin Chen, Hongbo Li, Guanxiang Liao, Xieping Dong

**Affiliations:** ^1^ Jiangxi University of Chinese Medicine, Nanchang, Jiangxi, China; ^2^ Department of Orthopaedics, JXHC Key Laboratory of Digital Orthopaedics, Jiangxi Provincial People’s Hospital, The First Affiliated Hospital of Nanchang Medical College, Nanchang, Jiangxi, China

**Keywords:** total hip arthroplasty, fused hip, 3D printing, tuberculosis, *Mycobacterium avium* infection

## Abstract

**Background:**

Converting a fused hip to total hip arthroplasty (THA) presents considerable surgical challenges. Traditional surgical techniques frequently result in malalignment of osteotomy planes and inaccurate prosthetic placement, leading to a high incidence of perioperative complications.

**Case summary:**

This report describes a 64-year-old male patient who underwent debridement surgery for right tuberculous hip arthritis 52 years ago. Following the operation, the joint gradually fused spontaneously, causing limited mobility. Over the past 4 years, the patient has experienced right knee pain and an unstable gait. The pathological fusion of the hip joint posed great challenges for THA, particularly in determining the osteotomy plane, ensuring adequate bone support, and reconstructing the acetabular rotation center. Using CT data, we designed and 3D printed a customized acetabular prosthesis with a series of combined guide systems, which allowed precise osteotomy and prosthetic implantation during the surgery. However, the patient developed a *Mycobacterium avium* infection following the procedure. As a result, debridement surgery, replacement of the acetabular liner, and combined pharmacological treatment were completed. Ultimately, the infection was controlled. A 5.5-year follow-up exhibited an improvement in the Harris hip score from 41 preoperatively to 90 postoperatively. The patient’s ability to perform activities of daily living improved significantly, and radiological follow-up indicated good prosthetic positioning without signs of loosening or displacement.

**Conclusion:**

The use of 3D-printed customized combined guides in THA offers a precise and safe treatment option for patients with hip joint fusion, effectively overcoming surgical challenges associated with altered anatomy. Moreover, this approach provides a reliable treatment reference for similar complex cases. The occurrence of *M. avium* infection in this patient underscores the importance of perioperative infection control and prompt management in patients with a history of tuberculosis.

## Introduction

Hip joint fusion can lead to functional impairment, resulting in a cascade of issues including lower back pain, knee pain, contralateral hip pain, and discrepancies in limb length, severely impacting the patient’s quality of life. An increasing number of patients are eager to alleviate the fused state of the hip joint and restore its function (Mimendia et al., 2024; Grappiolo et al., 2021). As a result, converting to total hip arthroplasty (THA) has been a feasible treatment choice, especially for younger patients with higher functional demands (Celiktas et al., 2017; Peng-Fei et al., 2019). However, converting a fused hip joint to THA presents significant technical challenges. The loss of normal anatomic landmarks following fusion and the absence of the femoral neck complicate the determination of the optimal osteotomy plane and precise placement of the acetabular prosthesis. This challenge is particularly pronounced in patients with a history of tuberculosis, as factors which include severe soft tissue contracture, osteoporosis, and potential infection significantly increase the surgical difficulty and perioperative complications (Tan and Chin, 2015).

This case report describes a patient who underwent THA 52 years after developing hip joint fusion owing to tuberculous arthritis. The surgery was successfully assisted by a 3D-printed customized combined guide. We present a detailed account of the surgical technique and the clinical outcomes of the follow-up, aiming to show the unique advantages of 3D printing technology in addressing the conversion from hip joint fusion to artificial joint replacement.

### Case presentation

#### General information

**FIGURE 1:**
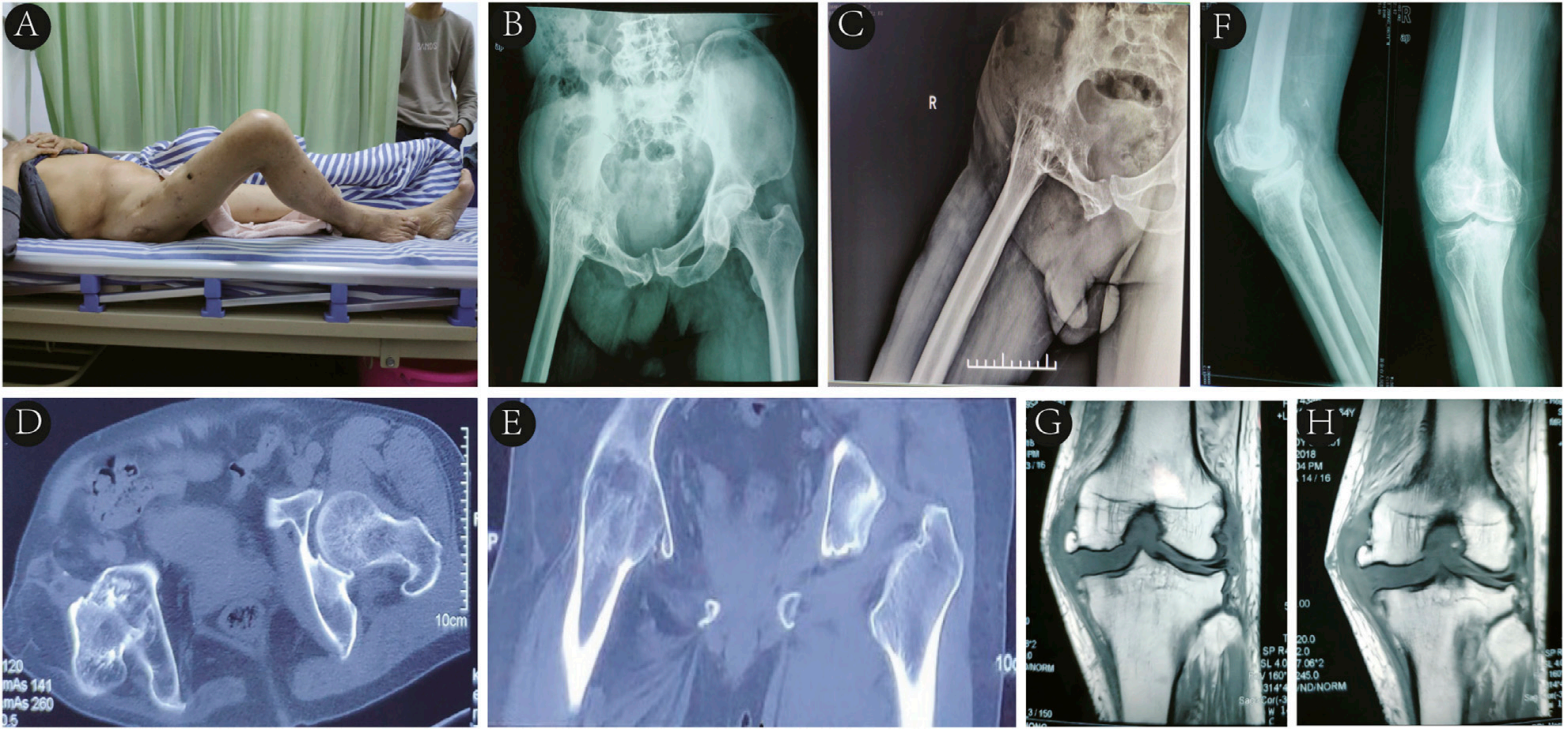
**(A)** Right hip joint fixed in 45° of flexion and 30° of abduction and external rotation. **(B–E)** Pelvic X-rays in standard position, lateral hip X-rays, and CT imaging revealing pelvic tilt, right hip ectopic fusion, and femoral neck near-total disappearance. **(F, G, H)** Right knee X-rays and MRI displaying degenerative changes, distal femoral and proximal tibial osteophyte formation, and lateral collateral ligament injury.

A 64-year-old male was admitted due to right hip joint fusion with limited mobility for 52 years and right knee pain with instability for the past 4 years. At the age of 12, he was diagnosed with tuberculous hip arthritis at a provincial children’s hospital and underwent thorough debridement of the tuberculous focus along with anti-tuberculosis drug therapy. Following this procedure, the right hip joint gradually fused with limited mobility, and there were no records of tuberculosis recurrence postoperatively. Over the last 4 years, he experienced worsening right knee pain and instability despite conservative treatment. The patient had well - controlled hypertension, with no significant personal or family history. Physical examination revealed an antalgic gait, with the right hip joint fixed in 45 degrees of flexion and 30 degrees of abduction and external rotation, as well as an 11 cm shortening of the right lower limb compared with the left (Figure 1A). The right knee joint exhibited varus deformity, with a positive medial stress test. However, sensation in both lower limbs and dorsalis pedis artery pulsation were normal. The Harris hip score was recorded at 41, and the Visual Analog Scale (VAS) score was 6. Laboratory tests indicated mild anemia (hemoglobin 110 g/L), increased erythrocyte sedimentation rate (24 mm/h), normal white blood cell counts and classifications, and slightly lowered albumin (39.2 g/L). Pelvic X-rays, lateral hip X-rays, and CT scans revealed pelvic tilt, ectopic fusion of the right hip joint, and near-total disappearance of the femoral neck (Figures 1B–E). X-ray and MRI of the right knee joint suggested degenerative changes, osteophyte formation at the distal femur and proximal tibia, and damage to the lateral collateral ligament (Figures 1F–H).

#### Therapeutic interventions

**FIGURE 2:**
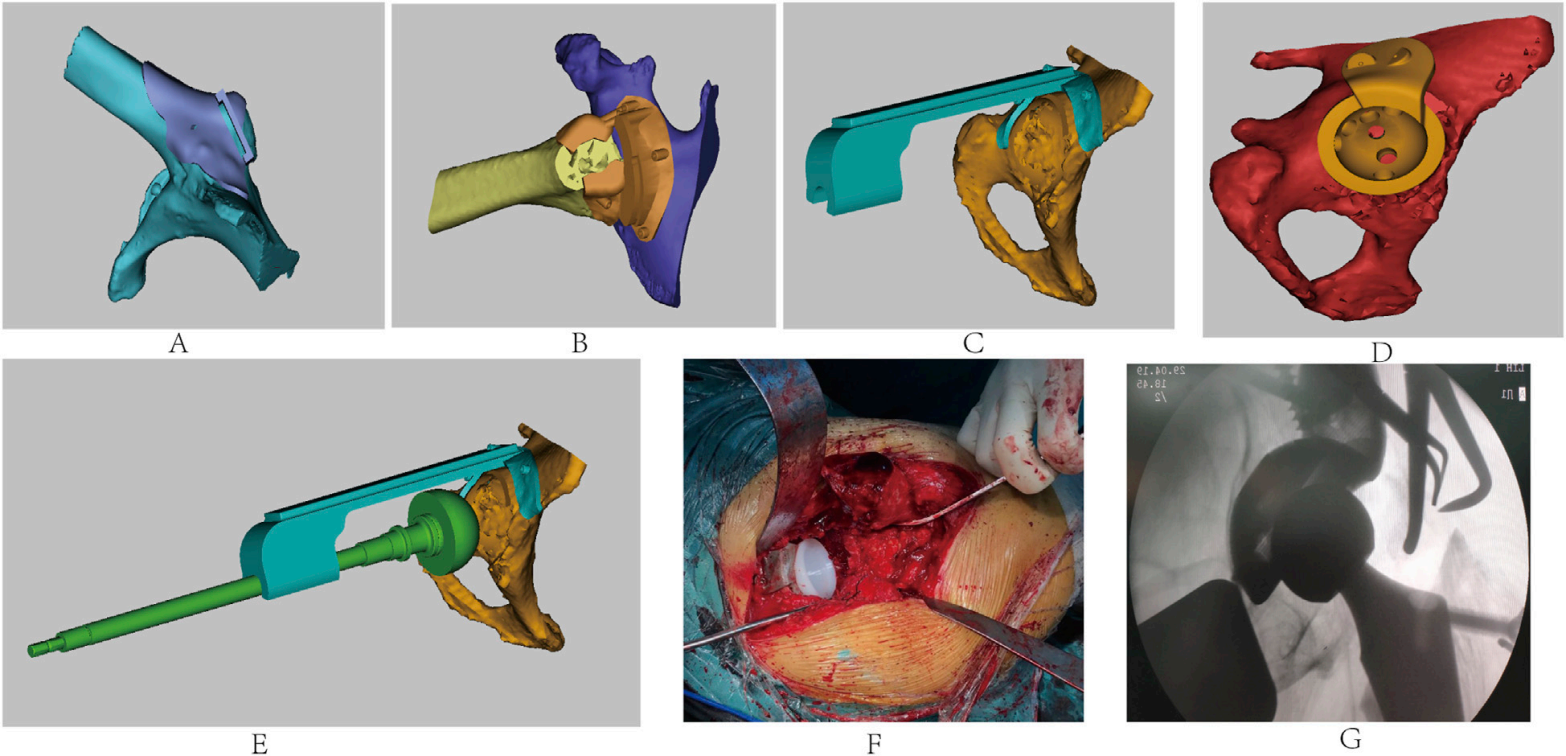
Design of the **(A)** greater trochanter osteotomy guide, **(B)** femoral neck osteotomy guide, **(C)** acetabular reamer guide, **(D)** personalized winged acetabular prosthesis, and **(E)** acetabular impacter. **(F)** Intraoperative procedures. **(G)** Intraoperative fluoroscopy showing good prosthetic positioning.

The patient’s childhood tuberculous hip arthritis led to the bony fusion of the hip, both the pelvis and femur were developmentally affected, resulting in the almost complete absence of the femoral neck and upward displacement of the hip joint center. Conventional THA techniques struggled to balance the bone stock distribution between the femoral and acetabular sides, making it difficult to estimate the bone stock available for prosthetic anchorage at both ends. This resulted in challenges in accurately locating the osteotomy plane and the original acetabulum, causing shifts in the center of rotation of the hip joint and affecting the stability of the prosthesis and the length and function of the affected limb. To address these challenges, we reconstructed 3D models of the pelvis and femur based on the patient’s CT data, designing a series of customized combined guides including a greater trochanter osteotomy guide (Figure 2A), a femoral neck osteotomy guide (Figure 2B), an acetabular reamer guide (Figure 2C), a customized winged acetabular implant (Figure 2D), and an acetabular impacter (Figure 2E). These guides allowed for the precise determination of the osteotomy plane and the acetabular rotation center, ensuring correct prosthetic placement.

Considering the need for femoral neck osteotomy dissection and abductor musculature preservation, we first designed the greater trochanter osteotomy guide. Then, based on its plane, we created the femoral neck osteotomy guide to balance bone stock on both sides. The acetabular reamer guide was designed using computer simulations to calculate the depth of acetabular reaming and the angle for placing the acetabular prosthesis. Finally, a porous surface winged acetabular cup and directed fixation screws were designed to accommodate the abnormally developed acetabulum, which enhanced immediate stability and promoted bone integration for permanent biological fixation. The surgery was performed through a standard posterior-lateral approach, extending along the original surgical incision, The fascia lata was thin but tense, with complete atrophy of the gluteus medius, gluteus maximus, and external rotator muscles, along with deformed greater trochanters and a shortened femoral neck resulting in complete fusion of the femoral head with the acetabulum. The 3D-printed personalized greater trochanter osteotomy guide was precisely aligned with the proximal femur to perform an accurate osteotomy, preserving the excised greater trochanter and attached abductors’ tendons. With the osteotomy surface as a reference, femoral neck osteotomy was performed under the guidance of the femoral neck osteotomy guide. Subsequently, the femoral head was removed. The right lower limb was internally rotated, with the plantar surface facing upward, and the acetabular reamer guide was secured to sequentially ream the acetabulum based on the preoperative plan. During this process, necrotic bone were discovered in the inferior-medial acetabulum, which were scraped out and sent for pathological examination, while the cavity was filled with bone cement.

We proceeded to implant the personalized porous surface acetabular cup with winged fixation screws, followed by the acetabular liner (Figure 2F). After typical preparations were made on the femoral side, the appropriately sized femoral component and head were implanted, with the hip joint being repositioned and evaluated for stability and range of motion. Then, the greater trochanter was repositioned and fixed with a K-wire. Intraoperative C-arm imaging indicated satisfactory prosthetic positioning (Figure 2G). The surgical site was flushed with saline, a drain was placed, and the incision was closed layer by layer.

## Results and follow-up

**FIGURE 3:**
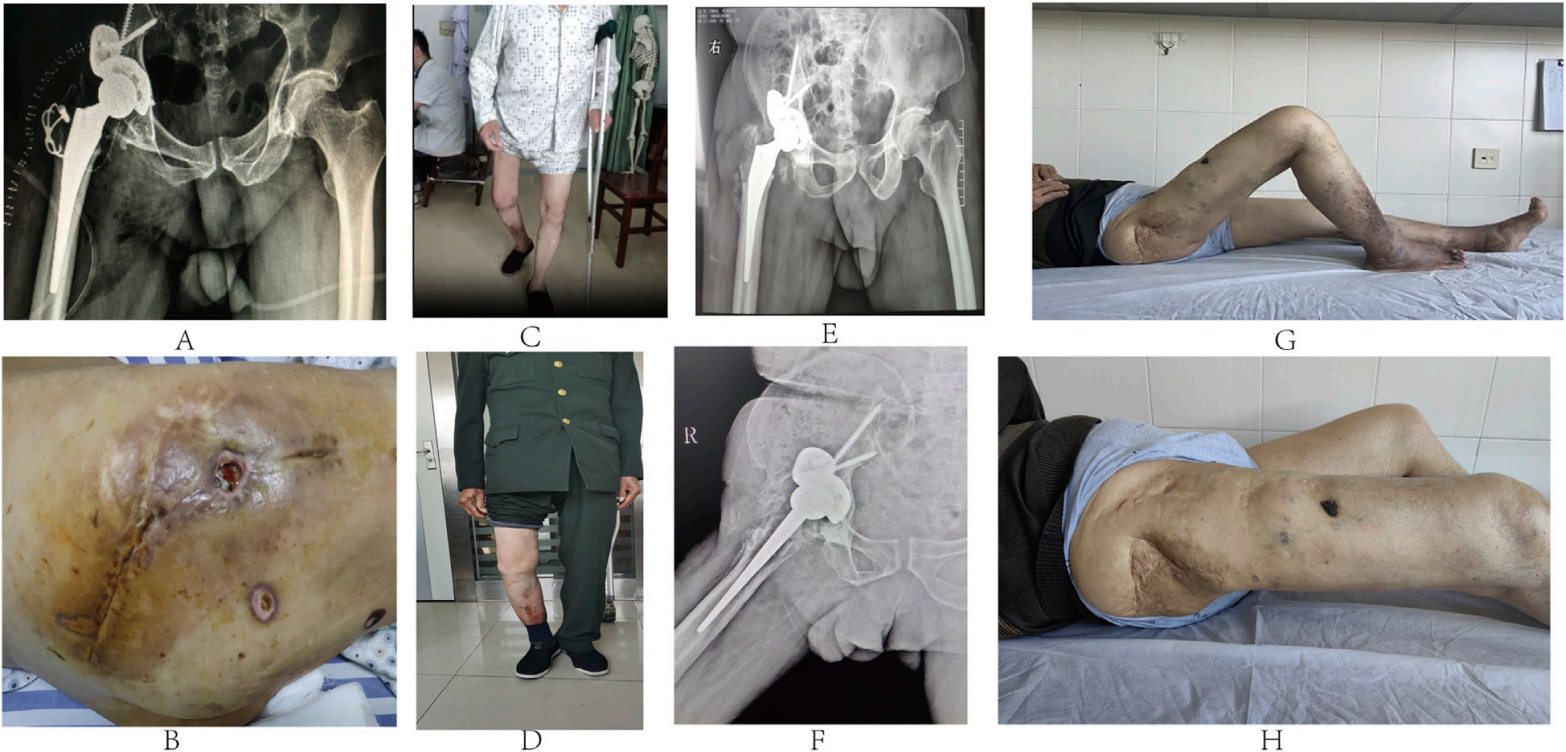
**(A)** Postoperative pelvic X-rays showing precise prosthesis positioning. **(B)** Ten days postoperatively, purulent discharge was noted from the incision. **(C)** Three months postoperatively, he walked with single crutch support. **(D)** He walked normally 5.5 years postoperatively. **(E, F)** At 5.5-year follow-up, pelvic X-rays in standard and lateral positions showing artificial joint stability without abnormalities. **(G, H)** Hip joint range of motion was 0–50° 5.5 years postoperatively.

Postoperatively, the patient received standard anti-infection treatment and was guided to perform rehabilitation exercises. Follow-up pelvic X-rays revealed the precise positioning of the prosthesis (Figure 3A). Pathological examination of the right hip suggested necrotic bone tissue and caseous necrosis, with negative acid-fast staining results. On the 10th postoperative day, the patient exhibited wound exudation and purulence (Figure 3B). Laboratory tests indicated low levels of red blood cells, hemoglobin, and platelets, along with mildly increased erythrocyte sedimentation rate (ESR), C-reactive protein (CRP), and procalcitonin. The T-SPOT tuberculosis infection test returned positive (score +). Multiple cultures of the purulent discharge yielded no growth of bacteria or fungi, and acid-fast staining did not exhibit the presence of *Mycobacterium tuberculosis*. However, *Mycobacterium avium* complex (MAC) was identified through DNA microarray chip analysis of the purulent fluid. The patient underwent a first-stage debridement surgery 40 days postoperatively to retain the prosthesis including replacement of acetabular liner and removal of K-wire fixation. Following the surgery, he received anti-infection drug therapy and symptomatic treatment, including platelet supplementation, blood transfusions, and gastric protection. The wound healed completely, and the patient was guided to perform non-weight-bearing walking exercises with the aid of crutches. At 3 months postoperatively, he was able to walk with single crutch support (Figure 3C), and by 1 year after the surgery, he was walking without assistance (Figure 3D). At the 5.5-year follow-up, he was walking normally, with pelvic X-ray examinations indicating stable prosthesis without abnormalities (Figures 3E, F). The Harris hip score improved from 41 preoperatively to 90 postoperatively, with the Visual Analog Scale (VAS) pain score decreasing from six preoperatively to one postoperatively. The range of motion of the hip joint was recorded as 0–50° (Figures 3G, H), and the right lower limb was 1 cm shorter than the left.

## Discussion

### Conversion of hip joint fusion to hip joint replacement

After hip joint fusion, although the joint may be stable and painless, long-term biomechanical changes can result in lower back pain, contralateral hip pain, ipsilateral knee pain, and other related issues. Most patients with hip joint fusion seek THA (Mimendia et al., 2024; Grappiolo et al., 2021; Whitehouse and Duncan, 2013). Literature indicates that converting hip joint fusion to THA yields good clinical results, including significant improvements in hip joint function, reductions in pain scores, and decreases in limb-length discrepancies (Lei et al., 2021; Zhan et al., 2024). For example, Flecher Xavier retrospectively explored 23 cases of THA using 3D-CT customized titanium alloy hydroxyapatite (HA)-coated femoral stems, reporting average Harris Hip Scores improving from 59 preoperatively to 89 postoperatively, with an average range of motion of 88°, a 62% reduction in low back pain, a 42% reduction in knee pain, and an average postoperative lower limb length discrepancy of 7.8 mm (Xavier et al., 2018). Similar to this literature, the present case achieved an increase in Harris hip score from 41 to 90, with hip joint motion from 0–50° and a reduction in limb-length discrepancy from 11 cm to 1 cm. However, due to anatomical abnormalities, fibrosis, and scarring of the surrounding soft tissues, traditional surgical methods often struggle to identify anatomical landmarks, determine osteotomy planes, and accurately place prosthetics in the original acetabulum. As a result, patients undergoing THA conversion post-fusion face greater technical challenges. Studies have indicated that the incidence of perioperative complications in patients undergoing THA conversion after hip fusion is significantly higher than that for primary THA, including risks of improper prosthetic placement, prolonged surgical duration, excessive intraoperative bleeding, high postoperative infection rates, and prosthetic loosening (Aderinto et al., 2012; Mariano et al., 2010; Mahla et al., 2023).

With the advancement of robotic technology and digital simulation-assisted techniques, some researchers have attempted to apply these new technologies in converting fused hips to THA. At first, Yi-he Hu reported a case where mixed reality combined with 3D printing technology assisted in the THA for a 59-year-old male patient with intertrochanteric femoral fracture and hip fusion, achieving precise acetabular cup placement while avoiding injury to the sciatic nerve (Peng-Fei et al., 2019). Adil SA reported on four bilateral and one unilateral THA procedures assisted by the Mako robotic arm in patients with ankylosing spondylitis-induced hip joint fusion. Compared with traditional THA, robotic-assisted surgery can simplify the surgical process and reduces perioperative complications (Syed Ali et al., 2021). Although these methods have exhibited good results in certain cases, surgeons need to undergo extensive training to master the technology, which presents a steep learning curve. Moreover, the high cost of robotic equipment and related consumables restricts its widespread application in many hospitals, especially in resource-limited settings. Additionally, the lack of long-term follow-up data means that the long-term effectiveness and potential late-stage complications of these new technologies remain uncertain.

In this case, the patient had a history of childhood tuberculous hip arthritis and underwent joint debridement surgery, contributing to spontaneous fusion of the hip joint and subsequent limitations in motion. Recently, he has experienced pain in the same-side knee and unstable walking, prompting the need for total knee arthroplasty while also requiring THA correction for right hip joint deformity. The patient encountered similar challenges as noted in the literature during the THA, specifically associated with determining the osteotomy plane and identifying the original acetabulum location. Therefore, computer-assisted techniques were utilized to reconstruct the hip joint model, design and 3D print a series of personalized combined guide systems and acetabular prostheses, successfully achieving precise osteotomy and prosthetic placement during the surgery. Detailed and precise preoperative planning is crucial for the success of the surgery and positive postoperative outcomes, as evidenced by the accurate placement of the prosthesis observed during the follow-up, and the patient reported satisfactory clinical outcomes.

### Prosthetic joint infection (PJI) after hip joint replacement

For patients with a history of hip joint tuberculosis, the potential for reinfection must be thoroughly considered prior to proceeding with THA. Most scholars agree that a resting period of at least 10 years following a tuberculosis infection is essential before joint replacement surgery can be safely performed (Neogi Devdatta et al., 2009). However, some researchers argue that such a prolonged waiting period may lead to significant functional impairment, adversely impacting the patient’s quality of life and work (Liangjun et al., 2016). While there is some contention regarding this matter, a general consensus suggests that a longer quiescent period is associated with a lower risk of postoperative recurrence. In this case, the patient had a 52-year quiescent period following a tuberculosis infection and did not experience any recurrence after THA.

However, this patient experienced a postoperative complication of PJI caused by MAC. After a first-stage thorough debridement involving the retention of the prosthesis and just the replacement of the liner, the infection was effectively managed. Literature suggests that MAC is one of the most common pathogenic non-tuberculous mycobacteria (NTM) species (Griffith David et al., 2007). Unfortunately, NTM infections are often ignored during the diagnostic process. Firstly, similar to most PJI patients, early NTM infections typically lack specific clinical symptoms, and may present with elevated serum biomarkers including CRP, ESR, D-dimer, and plasma fibrinogen (Rui et al., 2019). Furthermore, previous reports indicate that conventional cultures of NTM from tissue specimens can yield high negative rates, ranging from 36.3% to 61.5%, exhibiting their strong capacity for evasion (Zulipikaer et al., 2023). In this particular case, the patient underwent three purulent fluid cultures within 20 days of THA, all of which yielded no growth of bacteria or fungi, and showed negative acid-fast staining. It was not until strain identification through DNA microarray chip analysis that a diagnosis of *M. avium* infection was confirmed. Furthermore, this emphasizes that even when preoperative tests come back negative, physicians should maintain a high level of suspicion for postoperative infections and collect multiple samples of joint fluid and periprosthetic tissue for culture.

Given the patient’s history of tuberculosis, there are several potential sources of the MAC infection. One possibility is that the previous tuberculosis infection could have left the local tissue environment in a state of chronic inflammation or immune dysregulation. This altered tissue environment might have provided a more favorable niche for MAC colonization. For example, the scarred tissues from the tuberculosis-related inflammation could have had reduced blood supply and immune cell function, making it easier for MAC to establish an infection. Another potential source could be exogenous contamination during the surgical procedure. Operating rooms can be a source of various microorganisms, and if proper sterilization and aseptic techniques were not strictly adhered to, MAC could have been introduced into the surgical site. Additionally, the patient’s general health status and any potential comorbidities could have influenced their susceptibility to MAC infection. The mild anemia and slightly lowered albumin levels noted in the patient’s preoperative tests might have indicated a compromised immune system, making them more vulnerable to opportunistic infections like MAC. Moreover, tuberculosis remains highly prevalent in developing countries, where both *M. tuberculosis* and NTM can be positive in standard acid-fast staining tests. NTM are sometimes misidentified as *M. tuberculosis*, while NTM infections are resistant to anti-tuberculosis medications, necessitating careful differentiation between tuberculosis and non-tuberculous infections (Radha et al., 2020).

Recent studies suggest that the primary surgical approach for treating NTM-related PJI typically involves a first-stage removal of the prosthetic device with thorough debridement, followed by a second-stage revision surgery. Nevertheless, some researchers have posited that in cases where the implant fixation is secure and the infection is caused by non-resistant bacteria, a debridement and implant retention (DAIR) procedure may also be a viable option and has been successfully applied in some PJI cases (Eren et al., 2010). After confirming the MAC infection, a DAIR approach was employed, resulting in comprehensive debridement of necrotic and infected tissues, replacement of the acetabular liner, and postoperative combined anti-infection pharmacotherapy, with no recurrence of MAC observed during follow-up to date.

## Conclusion

In summary, treating patients who developed hip joint fusion due to tuberculosis in childhood through total hip arthroplasty (THA) is fraught with difficulties and has a high risk of perioperative complications. The 3D-printed personalized combined guide systems used in this study bring several distinct advantages. These guides, customized according to the patient’s unique CT-based anatomy, are far more precise than traditional methods in determining the osteotomy plane and acetabular rotation center. Moreover, the size of the acetabular reamer, as well as the direction and depth of reaming, can be guided by these customized plates, which greatly reduces the risk of misalignment-related complications such as prosthetic loosening. Compared with other similar research, the customized design of the guides in this study, like the specialized acetabular reamer guide, and the personalized design of the implant, such as the winged acetabular cup, can better adapt to the abnormal acetabulum and bone structure in patients with hip joint fusion. This not only improves the initial stability of the prosthesis but also promotes long - term bone integration, enhancing the overall prognosis of patients. However, the occurrence of *M. avium* infection in this patient serves as a strong reminder of the crucial need for strict perioperative infection control. Even with advanced surgical technology, overlooking infection prevention can lead to serious setbacks. In conclusion, the 3D-printed customized combined guide system offers a precise, safe, and effective treatment alternative for hip - joint - fusion patients. It overcomes many of the anatomical and technical challenges associated with such complex surgeries. Future research should concentrate on summarizing and improving the design techniques of 3D-printed personalized surgical guides and implants, and strengthening perioperative management. There should be a special emphasis on more effective infection - control strategies to achieve higher surgical success rates and significantly improve patients’ quality of life.

## Data Availability

The raw data supporting the conclusions of this article will be made available by the authors, without undue reservation.
